# Beyond hospice: the burden of palliative care needs in hospitals and long-term care facilities. A nationwide multicenter study

**DOI:** 10.1007/s40520-026-03445-w

**Published:** 2026-06-28

**Authors:** Graziano Onder, Alberto Zucchelli, Alba Malara, Gilda Borselli, Angela Iurlaro, Claudia Bauco, Angelo Bianchetti, Christian Bracco, Carmine Cafariello, Stefania Cartesio, Luca Cipriani, Giulia Deias, Diego De Leo, Elisa Durante, Gianpaolo Fortini, Fabio Gilioli, Stefania Giordano, Guido Horn, Myriam Macaluso, Lorenzo Palleschi, Elena Pinardi, Claudio Santini, Gianlorenzo Scaccabarozzi, Roberto Tarquini, Monica Torrini, Edoardo Varratta, Nicola Veronese, Maria Beatrice Zazzara, Dario Leosco, Giuseppe Bellelli, Palliative Care Day 2025 Working Group

**Affiliations:** 1 Università Cattolica del Sacro Cuore, Rome, Italy; 2 Fondazione Policlinico Gemelli IRCCS, Rome, Italy; 3 University of Brescia, Brescia, Italy; 4 Fondazione ANASTE Humanitas, Rome, Italy; 5 Società Italiana di Gerontologia e Geriatria, Florence, Italy; 6 ASL Frosinone, Frosinone, Italy; 7 Fondazione Teresa Camplani, Domus Salutis Brescia, Brescia, Italy; 8 Azienda Ospedaliera Santa Croce e Carle, Cuneo, Italy; 9 Provincia Romana Camilliani, Viterbo,, Italy; 10 ASL Roma 1, Rome, Italy; 11 Fondazione Sanità e Ricerca, Rome, Italy; 12 Griffith University, Gold Coast, Australia; 13 De Leo Fund, Padova, Italy; 14 Primorska University, Koper, Slovenia; 15 Gemelli Medical Center, Rome, Italy; 16 ASST Sette Laghi, Varese, Italy; 17 Ospedale S. Maria Bianca, AUSL Modena, Mirandola, MO Italy; 18 RSA Salus, Fondazione ANASTE Humanitas, Rome, Italy; 19 Fondazione Antea, Rome, Italy; 20 Azienda Ospedaliera San Giovanni-Addolorata, Rome, Italy; 21 Centro Studi Medicina della Complessità e Cure Palliative, Milano-Bicocca University, Monza, Italy; 22 Fondazione IRCCS San Gerardo, Monza, Italy; 23 Grassi Hospital, ASL Roma 3, Rome, Italy; 24 USL Toscana Centro, Empoli, FI Italy; 25 University of Florence, Azienda Ospedaliero Universitaria Careggi, Florence, Italy; 26 Faculty of Medicine, Saint Camillus University, Rome, Italy; 27 Unità Locale Socio Sanitaria 3 Serenissima, Venice, Italy; 28 Università degli Studi di Napoli Federico II, Naples, Italy

**Keywords:** Hospital, Long term care, Palliative care, Older adults

## Abstract

**Introduction:**

Palliative care is traditionally associated with hospice and cancer care, yet older adults admitted to hospitals and long-term care facilities (LTCF) frequently experience multimorbidity, frailty, and high symptom burden. We aimed to estimate the prevalence and characteristics of palliative care needs in Internal Medicine and Geriatrics hospital wards and LTCF in Italy.

**Methods:**

We conducted a national cross-sectional point-prevalence study. Adults aged ≥ 18 years hospitalized in participating wards or residing in LTCF were eligible. NECPAL 4.0 tool was used to identify patients and residents with palliative care needs. Additional indicators included functional and nutritional decline, repeated hospitalizations, dysphagia, pressure ulcers, delirium, and pain.

**Results:**

A total of 5,389 participants were included (3,303 hospital patients, median age 81 years; 2,086 LTCF residents, median age 86 years) from 235 facilities. Overall, 58.3% of hospitalized patients and 48.1% of LTCF residents had a positive NECPAL assessment. Prevalence was higher among patients with cancer (77.2% in hospital; 61.5% in LTCF), but remained substantial among those without cancer (50.8% in hospital; 46.8% in LTCF). Functional decline (56.2% hospital; 48.7% LTCF), nutritional decline (36.6%; 23.9%), dysphagia (17.6%; 28.8%), delirium (19.2%; 36.4%), and moderate-to-severe pain (25.9%; 20.3%) were common across settings.

**Conclusion:**

Palliative care needs are highly prevalent among hospitalized patients and LTCF residents and are not limited to oncology. These findings support systematic screening and integration of a needs-based palliative care approach within hospital and long-term care systems.

**Supplementary Information:**

The online version contains supplementary material available at https://doi.org/10.1007/s40520-026-03445-w.

## Key summary points

**Aim-** To estimate the prevalence of palliative care needs among patients admitted to Internal Medicine and Geriatrics hospital wards and among long term care facilities (LTCF) residents in Italy.

**Findings-** In this national point-prevalence study, 5,389 participants across 235 facilities, 58.3% of hospitalized patients and 48.1% of LTCF residents screened positive for palliative care needs according to the NECPAL tool. Although prevalence was highest among patients with cancer (77.2% in hospital; 61.5% in LTCF), a substantial proportion of individuals without cancer also screened positive (50.8% and 46.8%, respectively). Clinical indicators such as functional decline, nutritional deterioration, dysphagia, delirium, and moderate-to-severe pain were common.

**Message-** Palliative care needs are highly prevalent across acute and long-term care settings and extend beyond oncology, supporting systematic integration of a needs-based palliative care approach in hospital and long-term care populations.

**Supplementary Information:**

The online version contains supplementary material available at https://doi.org/10.1007/s40520-026-03445-w.

## Introduction

Integration of palliative care into the treatment pathways of patients with advanced chronic disease is associated with improvements in patients’ coping, overall well-being, and support networks, including family members and informal caregivers [1–3]. Beyond pain and symptom management, palliative care represents a comprehensive model that integrates physical, psychological, and social dimensions of care, addressing the needs of both patients with advanced chronic conditions and their families [4]. In addition to its clinical benefits, palliative care has been shown to improve healthcare resource utilization, for example by reducing hospital length of stay and overall treatment costs. As such, palliative care not only enhances quality of life advanced chronic diseases, but also contributes to a more sustainable and effective healthcare system [5, 6].

In many healthcare systems, palliative care is primarily delivered in hospice and home-based settings; however, hospitals and long-term care facilities (LTCF) play a complementary role in caring for vulnerable populations with advanced chronic and progressive diseases. Hospitals—particularly Geriatrics and Internal Medicine wards—frequently admit patients with multimorbidity, functional decline, and complex care needs, for whom disease-directed treatments alone may be insufficient to address symptom burden, psychosocial distress, and advance care planning needs [7–10]. These settings represent key points of care for patients who may benefit from a palliative approach, as they include persons across different disease trajectories and levels of clinical complexity, compared with disease-specific settings involving more selected patient groups. Similarly, LTCF care for residents with frailty, cognitive impairment, and advanced chronic conditions, who often experience prolonged decline and substantial, yet under-recognized, palliative care needs [11]. In these populations, the integration of palliative care is essential to improve quality of life, reduce symptom burden, and ensure goal-centered care ([12–13]).

A key component of this process is the appropriate identification of palliative care needs, including the assessment of symptom burden and broader aspects of patient well-being. Early recognition of palliative care needs has been associated with reduced hospital admissions and improved appropriateness of care, enabling timely and proportionate interventions aligned with individual care goals [14–16].

Despite this, palliative care needs in hospital and long-term care settings remain under-recognized, particularly among non-cancer patients, and data on their prevalence are limited. The aim of this study is to estimate the prevalence of palliative care needs among patients admitted to Internal Medicine and Geriatrics hospital wards and among LTCF residents in Italy, with the ultimate goal of increasing awareness among healthcare professionals and facilitating timely identification and appropriate management of these needs.

## Methods

### Study design

A point-prevalence study was conducted on the index date of November 11, 2025, in hospital wards of internal medicine and geriatrics and LTCF in Italy. LTCF in Italy include a range of long-term residential facilities providing integrated medical, nursing, and social care for people who cannot live independently. For the present study, we included facilities providing care to residents with moderate to high healthcare needs requiring regular nursing and medical supervision (known as R2), those delivering high-intensity care for severely dependent patients with complex conditions (R3), and facilities caring for residents with stable chronic conditions who primarily require long-term assistance with daily activities rather than intensive medical care (maintenance care).

Participating hospital wards and LTCF were recruited on a voluntary basis through national scientific societies and professional networks. The study invitation was disseminated through institutional communication channels and mailing lists. Members of these societies contributed to the dissemination of the initiative within their affiliated institutions, facilitating the participation of the centers in which they were clinically active. Although efforts were made to include centers from different regions, participation was voluntary and not based on systematic sampling; therefore, a formal response rate of healthcare facilities could not be calculated.

### Study sample

Participants (hospital patients and LTCF residents) were considered eligible for inclusion if they met all of the following criteria:

- Age ≥ 18 years;
- Hospitalized in participating hospital wards or residing in participating LTCF on November 11, 2025.
- Ability to sign informed consent or, if the participant was unable to provide consent, provision of signed authorization from a legally authorized representative.

All patients or residents present in participating wards and LTCFs on the index date who met eligibility criteria were considered for inclusion. Inclusion depended on the availability of informed consent, provided either directly by the participant or by a legally authorized representative. The total number of eligible patients was not systematically recorded.

### Questionnaire

The prevalence of palliative care needs was assessed using a study-specific questionnaire, implemented through a secure web-based platform developed using REDCap. The questionnaire was completed by study researchers, based on direct patient evaluation and review of clinical records. Prior to study initiation, study researchers received specific training on data collection procedures and on the use of the electronic case report form. Training also included guidance on the application and interpretation of the assessment tools and scales used in the questionnaire.

The questionnaire is structured into two sections.

The first section collects information related to the participating facility, including:

- Type of hospital ward (Internal Medicine or Geriatrics) for hospital settings, and type of LTCF;
- Availability of an internal Palliative Care service in hospitals, and the possibility of requesting consultation from a palliative care specialist in long-term care facilities.

The second section assesses the characteristics of enrolled patients/residents, including:

- Sociodemographic data (age, sex);
- Dysphagia, assessed based on presence for at least one month and type;
- Presence of pressure ulcers, including identification of advanced stages (III–IV);
- Indicators of palliative care needs assessed using the NECPAL 4.0 (Necesidades Paliativas) tool [14, 17–19]. The NECPAL instrument was developed and validated to promote timely, comprehensive, and integrated palliative care for individuals with advanced chronic diseases across healthcare and social care settings, in accordance with WHO recommendations. It has been applied across multiple care contexts, including primary care, hospital, intermediate care and nursing home settings, in populations with advanced chronic conditions and limited life prognosis [14, 17–19]. It’s structured into blocks:
  - Surprise Question (SQ): “Would I be surprised if this patient died in the next 12 months?”
  - Patient/family request or need indicator: Explicit demand for palliative care or perceived need by clinicians.
  - General clinical indicators: Functional decline (loss of > 30% in Barthel Index score over the previous 6 months), nutritional decline (unintentional loss of > 10% body weight over the previous 6 months), multimorbidity (presence of two or more advanced chronic conditions in addition to the primary disease), and repeated hospitalizations (≥ 2 hospital admissions within the last 12 months).
  - Disease-specific indicators: Indicators tailored to conditions.

NECPAL is considered positive (indicating presence of palliative care needs) when the Surprise Question is positive and at least one additional indicator is present, including the patient/family request or need indicator, general clinical indicators or disease-specific indicators.

- Pain assessment using the Numeric Pain Rating Scale (NPRS) or, for patients with advanced dementia unable to verbally communicate pain, the Pain Assessment in Advanced Dementia (PAINAD) scale [20–22]. The Numeric Pain Rating Scale (NPRS) is a unidimensional pain assessment tool ranging from 0 (no pain) to 10 (worst imaginable pain). The Pain Assessment in Advanced Dementia (PAINAD) scale is specifically designed for patients with advanced dementia who are unable to verbally communicate pain. It assesses five domains (breathing, vocalization, facial expression, body language, and consolability), each scored from 0 to 2, with higher scores indicating greater pain severity.
- Delirium screening using the 4AT tool [23]. The 4AT is a rapid screening tool for cognitive impairment and delirium and is one of the most extensively validated delirium assessment instruments in the scientific literature. A score ≥ 4, although not diagnostic, suggests the presence of delirium.

The selected clinical indicators were not intended to provide a comprehensive assessment of all distressing symptoms. Rather, they were chosen as clinically relevant and easily standardizable markers of vulnerability and clinical complexity, suitable for a point-prevalence design and feasible across multiple care settings.

### Sample and statistical analysis

As this is a descriptive study, a formal sample size calculation was not performed. However, based on the number of participating centers and in line with similar previous experiences, such as Delirium Day [24] and Prescription Day [25], we estimate the recruitment of approximately 5,000 participants, including 3,000 patients in the hospital setting and 2,000 in the LTCF setting.

Categorical variables are reported as absolute numbers and percentages. Continuous variables are presented as mean ± standard deviation or median with interquartile range, as appropriate. To compare characteristics of participants according to NECPAL positivity, we used Chi-square test for categorical variables. These comparisons were exploratory and no multilevel or multivariable analyses were performed, in line with the primarily descriptive aim of the study. Statistical analyses were performed using IBM SPSS Statistics version 30 (IBM Corp., Armonk, NY, USA).

### Ethics committee approval

Ethical approval was obtained from the Italian National Ethics Committee for Public Research Bodies (CEN). Written informed consent for participation in the study and for the processing of personal data, was obtained either directly or through a family member, caregiver, or legal representative.

## Results

### Characteristics of participating centers and enrolled patients/residents

A total of 235 care facilities participated in the study, including 156 hospital wards and 79 LTCFs, with a total of 5,524 patients/residents enrolled. Participants with missing data on age (*n* = 6; 0.1% of the study sample) or an incomplete NECPAL assessment (*n* = 129; 2.3%) were excluded, resulting in a final sample of 5,389 participants. As shown in Table 1, an internal palliative care service or the possibility to request specialist consultation was available in 62.8% of hospital wards and 64.6% of LTCFs. The final study sample consisted of 3,303 hospitalized patients and 2,086 LTCF residents. The median age was higher among LTCF residents compared with hospitalized patients (86 years, IQR 80–91 vs. 81 years, IQR 73–87), and women were more prevalent in LTCF (70.9% vs. 48.8%). Dementia was markedly more frequent among LTCF residents (72.2%) than hospital patients (28.6%), whereas cancer (28.2% vs. 8.7%) and heart disease (41.1% vs. 26.4%) were more common in hospital settings (Table 1).

Within hospital settings, 100 Internal Medicine wards and 56 Geriatric wards participated in the study. The availability of palliative care services was similar (62.0% vs. 64.3%). Patients admitted to Geriatric wards were older (median age 85 vs. 79 years) and more frequently female (52.5% vs. 46.9%). Dementia and cerebrovascular disease were more prevalent in Geriatric wards, whereas cancer was more frequent in Internal Medicine wards (Table 1a).

### Analysis of NECPAL tool

**Fig. 1:**
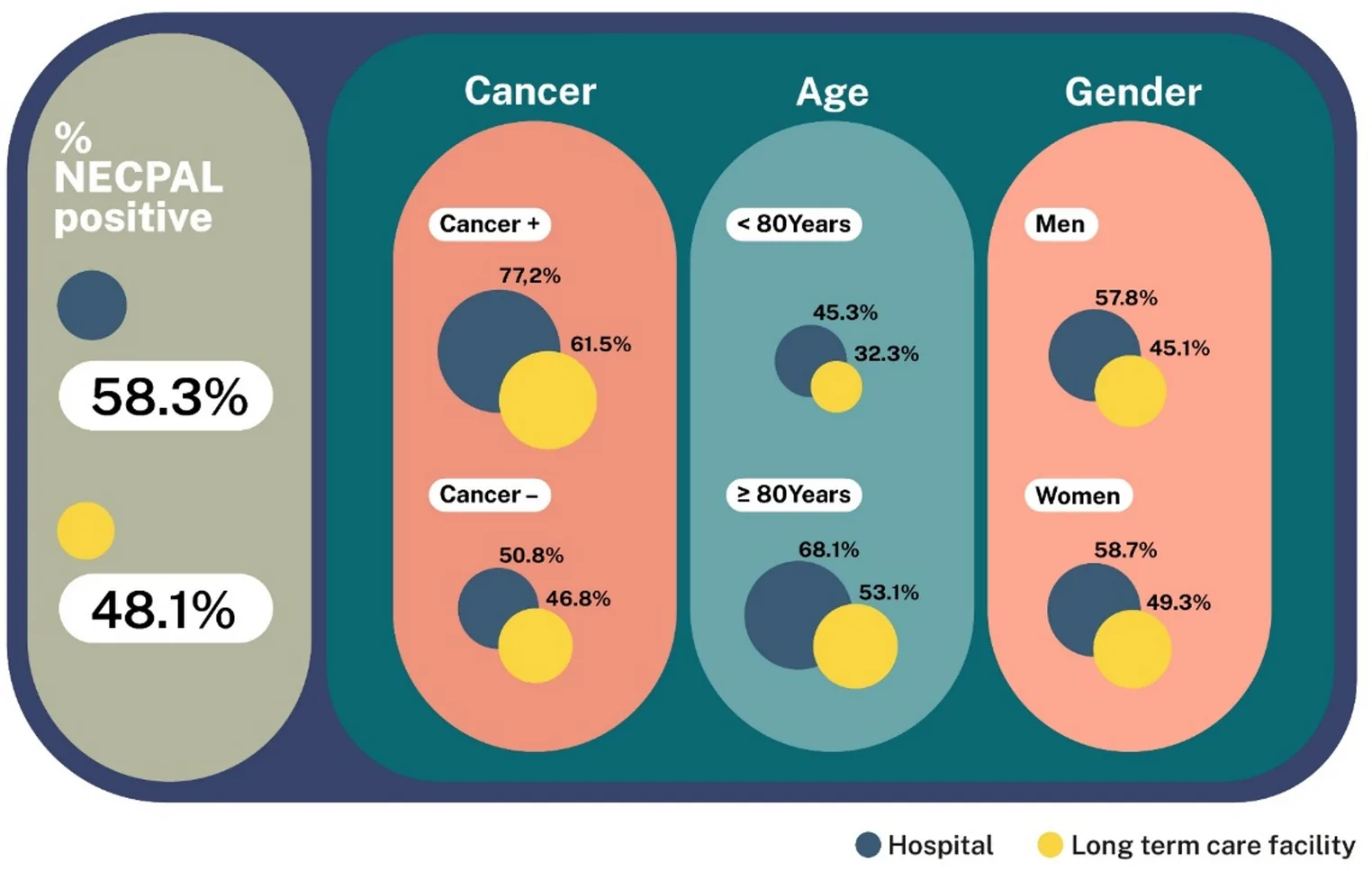
Descriptive representation of positive NECPAL assessment in hospital patients and long term care facilities residents

Positive NECPAL result was observed in 58.3% of the overall hospital population and in 48.1% of LTCF residents. As shown in Fig. 1, positive NECPAL result was consistently higher among older participants, individuals with cancer, and women across both hospital and LTCF settings. Within the hospital setting, positive NECPAL assessment was observed in 68.1% of patients aged ≥ 80 years compared with 45.3% of those aged < 80 years (*p* < 0.001), while in LTCF the corresponding prevalence was 53.1% and 32.3%, respectively (*p* < 0.001). Similarly, patients and residents with cancer showed substantially higher prevalence of positive NECPAL compared with those without cancer, both in hospitals (77.2% vs. 50.8%, *p* < 0.001) and LTCF (61.5% vs. 46.8%, *p* < 0.001). NECPAL positivity was similar in men and women in both settings, with prevalence rates of 58.7% versus 57.8% in hospitals (*p* = 0.60) and 49.3% versus 45.1% in LTCF (*p* = 0.09). Overall, the prevalence of positive NECPAL assessment remained consistently higher in hospital patients than in LTCF residents across all subgroups.

Within the hospital setting, the prevalence of NECPAL positivity was consistent across types of wards (Table 2a). When adopting more stringent thresholds for identifying palliative needs, 55.6% of the total hospital population met the criteria for a positive Surprise Question combined with at least two indicators. This proportion adjusted to 49.5% for at least three indicators and 39.0% for at least four indicators. A similar trend was observed among LTCF residents, with prevalence rates shifting from 44.9% (two indicators) to 33.4% (three indicators) and 22.1% (four or more indicators).

### Palliative care needs

Clinical indicators of palliative care needs were common in both hospitalized patients and LTCF residents (Table 2). Functional decline was the most frequent indicator in both settings, while dysphagia and delirium were more common among LTCF residents. Nutritional decline, repeated hospitalizations, and pressure ulcers were more frequently observed in hospitalized patients. Moderate-to-severe pain was also common in both groups. Within hospital wards, dysphagia, pressure ulcers, functional decline, nutritional decline and delirium were most frequently observed in patients admitted to Geriatric wards compared with those admitted to Internal Medicine wards. Repeated hospitalizations and moderate to severe pain were more frequent in Internal Medicine wards (Table 3a).

## Discussion

This large multicenter point-prevalence study, conducted across 235 facilities and including 5,389 participants, shows a high prevalence of palliative care needs in both geriatric and internal medicine hospital wards and LTCF. More than half of hospitalized patients (58.3%) and nearly half of LTCF residents (48.1%) screened positive according to NECPAL criteria.

NECPAL positivity should not be interpreted as a proxy for short-term mortality. The NECPAL instrument was developed to support early identification of patients with advanced chronic conditions who may benefit from a palliative care approach, combining the Surprise Question with general and disease-specific clinical indicators rather than relying exclusively on prognosis [14, 26]. Therefore, a positive result reflects the presence of multidimensional vulnerability and progressive clinical complexity. Importantly, although participants were not selected on the basis of specific diseases or prognostic criteria, NECPAL assessment was conducted among individuals admitted to acute care hospitals and long-term care facilities rather than in the general population. These settings are characterized by a high burden of chronic illness, functional impairment, and clinical complexity, making NECPAL an appropriate tool for studying palliative care needs and identifying patients who may benefit from a palliative care approach in these populations.

The distribution of indicators in our cohort supports this interpretation. In hospitalized patients, high rates of functional decline (56.2%), nutritional deterioration (36.6%) and repeated hospitalizations (45.3%) describe unstable trajectories. Markers of frailty such as dysphagia (17.6%), pressure ulcers (14.9%), and delirium (19.2%) were frequent, alongside substantial symptom burden, with about half reporting pain and 25.9% describing moderate-to-severe intensity. A comparable pattern emerged among LTCF residents, characterized by functional decline, dysphagia, delirium and clinically significant pain. Overall, these findings indicate that palliative care needs are structurally embedded in contemporary hospital and long-term care populations [27].

Our results are consistent with prior evidence documenting high levels of palliative care needs among hospital patients and LTCF residents. In long-term care, the SHELTER study demonstrated that LTCF residents across Europe present substantial disability, geriatric syndromes and complex care needs [11]. Although not designed as a palliative screening study, SHELTER clearly outlined the epidemiological substrate that underpins significant palliative care demand in this population. More recently, qualitative work by Cole et al. [28] identified key domains triggering palliative care referral in LTCF and highlighted the absence of standardized and validated referral criteria. Similarly, Carpenter et al. [29] documented a high prevalence of pain, psychological distress, and informational needs among older adults admitted to skilled nursing facilities, underscoring the under-recognition of palliative needs in post-acute care, although their sample was limited. In studies specifically using validated screening tools such as NECPAL in the hospital setting, the prevalence of palliative care needs appears consistently high. In acute hospital populations, approximately 27–28% of patients have been identified as NECPAL-positive, while in critically ill populations this proportion may reach around one-third of patients ([30–31]). These differences likely reflect heterogeneity in case-mix, disease severity, and care environments.

**Table 1:** Characteristics of participating centers and hospital patients/long term care facilities residents

	Hospital	Long-term care facility
**Ward/facility characteristics**		
N of participating wards/facilities	156	79
Availability of Palliative Care service/consultation (%)	98 (62.8%)	51 (64.6%)
**Patients/residents characteristics**		
N of participating patients/residents	3,303	2,086
Age, median (IQR)	81 (73–87)	86 (80–91)
Female gender (%)	1,613 (48.8%)	1,479 (70.9%)
Heart disease (%)	1,357 (41.1%)	551 (26.4%)
Cancer (%)	931 (28.2%)	182 (8.7%)
Cerebrovascular disease (%)	574 (17.4%)	534 (25.6%)
Dementia (%)	945 (28.6%)	1,507 (72.2%)
Other neurodegenerative disease (%)	178 (5.4%)	197 (9.4%)
Diabetes (%)	878 (26.6%)	439 (21.0%)
Pulmonary disease (%)	814 (24.6%)	312 (15.0%)
Liver disease (%)	255 (7.7%)	105 (5.0%)
Peripheral vascular disease (%)	425 (12.9%)	140 (6.7%)

IQR=InterQuartile Range

**Table 2:** Palliative care needs in enrolled patients/residents

	Hospital *n* = 3,303 (%)	Long-term care facility *n* = 2,086 (%)
Dysphagia	579 (17.6%)	596 (28.8%)
Any pressure ulcer	484 (14.9%)	205 (9.9%)
Pressure ulcer (stage III-IV)	149 (4.5%)	59 (2.8%)
Functional decline*	1,855 (56.2%)	1,015 (48.7%)
Nutritional decline¶	1,209 (36.6%)	498 (23.9%)
Repeated hospitalizations§	1,497 (45.3%)	221 (10.6%)
Delirium #	595 (19.2%)	570 (36.4%)
Any pain¥	1,590 (48.1%)	1,031 (49.4%)
Moderate to severe pain¥	856 (25.9%)	424 (20.3%)

* loss of > 30% in Barthel Index score over the previous 6 months

¶ unintentional loss of > 10% body weight over the previous 6 months

§ ≥2 hospital admissions within the last 12 months

# 4AT score ≥ 4. Data on delirium were available for 3,105 patients in hospital wards (94.0% of study sample) and 1,564 long term care facilities residents (75.0%)

¥ Any pain is defined as Numeric Pain Rating Scale ≥ 1 or Pain Assessment in Advanced Dementia ≥ 1; moderate to severe pain is defined as Numeric Pain Rating Scale ≥ 4 or Pain Assessment in Advanced Dementia ≥ 4

A key finding of our study is that palliative care needs are not limited to oncology. Although cancer patients showed high NECPAL positivity, a substantial proportion of individuals without cancer also met objective criteria for palliative care needs. This observation aligns with comparative research demonstrating that patients with advanced heart failure, COPD, dementia and other chronic conditions experience symptom burden and care complexity comparable to cancer populations [32]. Furthermore, hospitalized patients with end-stage non-cancer diseases show physical and psychosocial needs similar to those of terminal cancer patients [33], and community-based studies have documented persistent unmet needs among elderly non-cancer patients receiving home care [34].

Taken together, the available evidence supports a shift from a diagnosis-based to a needs-based referral model. In aging societies characterized by chronic organ failure, frailty and multimorbidity, suffering and complexity are driven by trajectory of decline rather than diagnostic label. Restricting access to palliative expertise based primarily on malignancy risks systematic under-recognition of vulnerable populations.

Our findings have direct implications for health system organization. Palliative care should not be confined to hospice or late-stage contexts. As emphasized by Kelley and Morrison [27], palliative care is an interdisciplinary approach aimed at improving quality of life for patients with serious illness at any stage, whereas hospice represents a specific model focused on end-of-life care. Evidence from hospital settings shows that structured integration of palliative care improves symptom control, enhances communication, and reduces the use of potentially non-beneficial intensive treatments. ([35–36]) In LTCF, the presence of palliative consultation has been associated with reductions in end-of-life hospitalizations, supporting the value of specialist input in long-term care environments [37]. However, identification alone is insufficient. Implementation requires systematic screening processes, professional training and integration pathways. Barriers such as prognostic uncertainty and limited use of structured tools have been documented in primary care and LTCF settings, highlighting the need for organizational strategies to translate epidemiological burden into effective care delivery.

Main limitation of the present study refers to the fact that it is not nationally representative, which limits the generalizability of the results. As facility participation was voluntary, institutions more engaged in palliative care may have been more likely to participate, potentially resulting in an overestimation of the prevalence of identified needs. In addition, the point-prevalence design, based on data collection performed on a single index day, may have been influenced by temporal factors such as fluctuations in admissions, clinical instability, or seasonal patterns, which could also have affected prevalence estimates.

The 4AT assessment was missing in about one-fourth of the LTCF sample. As the reasons for missing data were not systematically collected, the evaluation of delirium in this subgroup may be limited. Finally, exclusion of individuals unable to provide informed consent may have resulted in an underestimation of the true prevalence and severity of palliative care needs, as more vulnerable or severely ill patients were likely not included. The main strengths of this study are its large multicenter design and the use of validated multidimensional assessment tools (NECPAL, 4AT, and PAINAD). These elements strengthen the methodological quality of the study and allow meaningful comparisons across different care settings.

## Conclusions

The present study shows that palliative care needs are highly prevalent in both acute hospital wards and LTCF and affect patients across diagnostic categories. Approximately half of individuals in these settings present objective indicators of multidimensional vulnerability and progressive decline. These findings support the systematic integration of a needs-based palliative care approach within both hospital and long-term care systems. The high prevalence of multidimensional vulnerability observed in our cohort indicates that palliative care needs are common and predictable in these settings [12]. Our findings have direct implications for health system organization: palliative care should not be confined to hospice but must become a co-managed interdisciplinary approach aimed at improving quality of life at any stage of advanced illness.

## Supplementary Information

Below is the link to the electronic supplementary material.

Supplementary Material 1

## Data Availability

No datasets were generated or analysed during the current study.
