# Supplementary material for: Beyond hospice: the burden of palliative care needs in hospitals and long-term care facilities. A nationwide multicenter study
Source: Aging Clin Exp Res. 2026 Jun 28;38(1):193. doi: 10.1007/s40520-026-03445-w (PMC13582221; doi:10.1007/s40520-026-03445-w)
Supplement: Supplementary file 1 [file 40520_2026_3445_MOESM1_ESM.docx]

**APPENDIX**

**PALLIATIVE CARE DAY 2025 WORKING GROUP MEMBERS (in alphabetical order)**

**Irene Abatangelo** (Ospedale San Leopoldo Mandic, Merate/LC), **Irene Abati** (CRA I Lecci, Parma), **Rosa Abbruzzese** (Ospedale Cardinal Massaia, ASL di Asti), **Desirée Addesi** (Azienda Ospedaliero Universitaria Renato Dulbecco, Catanzaro), **Roberto Agarinis** (Presidio Ospedaliero di San Daniele/UD), **Romina Alessandroni** (Ospedale Santa Maria della Pietà, Camerino/MC), **Manuela Alessio** (Ospedale Michele e Pietro Ferrero, Verduno/CN), **Elena Allocca** (Istituto per la Sicurezza Sociale, San Marino/RSM), **Chiara Aloise** (ASST Grande Ospedale Metropolitano Niguarda, Milano), **Giulia Amadio** (Ospedale San Valentino, Montebelluna/TV), **Angelo Andina** (Residenza Don Maurizio Masseroni, Bellinzago Novarese/NO), **Elisabetta Anelito** (University of Florence), **Anna Grazia Angeletti** (Ospedale del Delta, Lagosanto/FE), **Beatrice Angelieri** (RSA Madonna delle Grazie, Filadelfia/VV), **Alessandra Annaloro** (Ospedale San Carlo Borromeo, Milano), **Marco Gianpiero Antivalle** (Casa di Cura Beato Luigi Talamoni, Lecco), **Raffaele Antonelli Incalzi** (Fondazione Policlinico Universitario Campus Bio-Medico, Roma), **Alessandra Anzuini** (Ospedale Alessandro Manzoni, Lecco), **Romeo Aracri** (RSA Madonna delle Grazie, Filadelfia/VV), **Lucia Argento** (RSA Coteto, Livorno), **Giacomo Argiolas** (ARNAS Ospedale G. Brotzu, Cagliari), **Giuseppe Armentaro** (Azienda Ospedaliero Universitaria Renato Dulbecco, Catanzaro), **Laura Arosio** (Ospedale San Leopoldo Mandic, Merate/LC), **Alessandra Arrigoni** (Presidio Ospedaliero di Gardone Val Trompia/BS), **Eleonora Assanto** (Azienda Ospedaliera San Giovanni Addolorata, Roma), **Sebastiana Atzori** (Azienda Ospedaliero Universitaria di Sassari), **Jane Gianira Avendano Olivares** (Fondazione Istituto Geriatrico La Pelucca Onlus, Sesto San Giovanni/MI), **Gabriela Avram** (Centro Servizi Contarini, Mestre/VE), **Barbara Babbato** (Residenza Riviera del Brenta, Dolo/VE), **Herman Bacoli** (RSA Villa Torano, Torano Castello/CS), **Tullia Baietto** (Presidio Ospedaliero Beata Vergine Consolata Fatebenefratelli, San Maurizio Canavese/TO), **Luisa Balcet** (Presidio Ospedaliero Beata Vergine Consolata Fatebenefratelli, San Maurizio Canavese/TO), **Giulia Ballarin** (Centro Servizi Anziani Felice Federico Casson, Chioggia/VE), **Pietro Ballesini** (Azienda Ospedaliero-Universitaria di Modena), **Francesco Baratta** (Azienda Ospedaliero-Universitaria Policlinico Umberto I, Roma), **Barbara Barbato** (Ospedale San Valentino, Montebelluna/TV), **Luciano Barbazza** (Centro Servizi San Giobbe, Venezia), **Silvia Barigazzi** (Ospedale Luigi Sacco, Milano), **Giacomo Baron** (Ospedale Policlinico G.B. Rossi, Verona), **Maria Lucia de Lourdes Barrionuevo** (RSA Villa San Clemente, Villasanta/MB), **Giorgio Basile** (Azienda Ospedaliera Universitaria Gaetano Martino, Messina), **Francesca Battaggia** (Centro Servizi per Anziani Nazaret, Zelarino/VE), **Giuseppe Battaglia** (RSA Madonna delle Grazie, Filadelfia/VV), **Cesare Battegazzore** (RSA Villa delle Rose, Ponte Nizza/PV), **Claudia Bauco** (Ospedale Santa Scolastica, Cassino/FR), **Mirko Beccalli** (Ospedale San Carlo Borromeo, Milano), **Liliana Bejinariu** (Residenza al Castello, Alpignano/TO), **Maristella Belfiori** (Azienda Socio-sanitaria Locale di Cagliari), **Giuseppe Bellelli** (Fondazione IRCCS San Gerardo dei Tintori, Monza), **Federica Bellone** (Azienda Ospedaliera Universitaria Gaetano Martino, Messina), **Angela Belsito** (RSA Villa Torano, Torano Castello/CS), **Valeria Benaglia** (IRCCS Istituti Clinici Scientifici Maugeri, Milano), **Giovanni Benanti** (IRCCS Ospedale San Raffaele, Milano), **Leonardo Bencivenga** (Azienda Ospedaliera Universitaria Federico II, Napoli), **Valentina Benedetti** (Ospedale Regina Montis Regalis, Mondovì/CN), **Vincenza Beneduce** (Ospedale San Bassiano, Bassano del Grappa/VI), **Cristina Benetazzo** (Ospedali Riuniti Padova Sud, Monselice/PD), **Alberto Benetti** (ASST Grande Ospedale Metropolitano Niguarda, Milano), **Eliana Berardi** (Ospedale Civile di Sondrio), **Filippo Bergamo** (Centro Servizi Contarini, Mestre/VE), **Andrea Bernardini** (RSA di Cascia/PG), **Giulia Bertaccini** (Ospedale G.B. Morgagni - L. Pierantoni, Forlì), **Laura Bertarini** (Azienda Ospedaliero-Universitaria di Modena), **Andrea Berti** (Casa di Cura Villa Immacolata, Viterbo), **Serena Bertolio** (Ospedale Santa Chiara, Trento), **Marco Bertolotti** (Ospedale Civile Sant'Agostino Estense, Baggiovara/MO), **Cristina Bettinsoli** (RSA Madre Teresa di Calcutta, Brescia), **Giovanna Bianchi** (Presidio Ospedaliero di Gardone Val Trompia/BS), **Anna Bianco** (Arcispedale Santa Maria Nuova, Reggio Emilia), **Gianluca Bianco** (Presidio Ospedaliero di Montichiari/BS), **Chiara Bicego** (Centro Servizi Sociali Villa Serena - Residenza Fontanelle di Trissino/VI, Residenza Giardino di Recoaro Terme/VI, Villa Serena di Valdagno/VI), **Adelaide Biondaro** (RSA AltaVita IRA, Padova), **Carolina Biondi** (RSA Coteto, Livorno), **Michela Bizzi** (Residenza Protetta Chiara Luce, Casarza Ligure/GE), **Mario Bo** (Azienda Ospedaliero-Universitaria Città della Salute e della Scienza di Torino), **Stefano Boffelli** (Ospedale Fondazione Poliambulanza, Brescia), **Carolina Bologna** (AORN Sant'Anna e San Sebastiano, Caserta), **Martina Bon** (Ospedale Santa Maria della Misericordia, Rovigo), **Antonio Bonaiuto** (Azienda Ospedaliera Universitaria Gaetano Martino, Messina), **Sara Bonardi** (Istituto Ospitale Magri Onlus, Urgnano/BG), **Alessandro Bondioni** (Azienda Socio Sanitaria Territoriale degli Spedali Civili di Brescia), **Martina Bonetto** (Azienda Ospedaliera Sante Croce e Carle, Cuneo), **Nadia Bongiorni** (Istituto Geriatrico Piero Redaelli - ASP Golgi Redaelli, Milano), **Elisa Boni** (RSA Cottolengo, Fornacette/PI), **Stefano Boni** (Ospedale degli Infermi, Faenza/RA), **Andrea Bonini** (Ospedale San Leopoldo Mandic, Merate/LC), **Giulia Bonomessi** (Ospedale Civile di Cuorgnè/TO), **Marta Boscolo** (Casa di Cura Sileno e Anna Rizzola, San Donà di Piave/VE), **Elena Boscolo Fiore** (Centro Servizi Anziani Felice Federico Casson, Chioggia/VE), **Giulia Bosio** (Azienda Socio Sanitaria Territoriale degli Spedali Civili di Brescia), **Claudia Bozzini** (Ospedale Policlinico G.B. Rossi, Verona), **Christian Bracco** (Azienda Ospedaliera Sante Croce e Carle, Cuneo), **Tiziana Brambati** (Ospedale Santissima Annunziata, Savigliano/CN), **Alice Silvia Brera** (Fondazione IRCCS Policlinico San Matteo, Pavia), **Silvia Brogna** (Ospedale San Bassiano, Bassano del Grappa/VI), **Antonio Brucato** (Ospedale Fatebenefratelli, Milano), **Jessica Brunetti** (RSA di Cascia/PG), **Rosamaria Bruni** (Ospedale Giovanni Paolo I, Agordo/BL), **Marta Brusati** (Residenza Don Maurizio Masseroni, Bellinzago Novarese/NO), **Raffaella Buggio** (RSA AltaVita IRA, Padova), **Ester Bukli** (Ospedale Santa Maria della Misericordia, Rovigo), **Matteo Bulgaresi** (Ospedale Santa Maria Annunziata, Bagno a Ripoli/FI), **Giuseppe Burattin** (Ospedale Santa Maria della Misericordia, Rovigo), **Maria Burdino** (RSA Villa Elisabetta, Cortale/CZ), **Michela Emma Burlone** (Azienda Ospedaliero-Universitaria Maggiore della Carità, Novara), **Sandra Buscaglia** (Ospedale San Paolo, Savona), **Michela Cadeddu** (Ospedale Sirai, Carbonia/SU), **Sara Calamusa** (RSA Salus, Roma), **Marco Caldarone** (Ospedale Cardinal Massaia, ASL di Asti), **Elisa Calistri** (University of Florence)**, Greta Campanale** (Ospedale Giovanni XXIII - Policlinico di Bari), **Martina Campi** (IRCCS Ospedale Policlinico San Martino, Genova), **Marta Canesi** (Fondazione IRCCS San Gerardo dei Tintori, Monza), **Claudia Cantini** (Ospedale San Jacopo, Pistoia), **Mirella Capano** (RSA Santa Maria del Monte, Petrizzi/CZ), **Carla Capasso** (Fondazione Casa di Industria Onlus, Brescia), **Sara Capellano** (Ospedale San Paolo, Savona), **Ivonne Capelli** (Casa Protetta Villa Giulia, Bologna), **Roberta Capelli** (Policlinico di Sant'Orsola - IRCCS Azienda Ospedaliero-Universitaria di Bologna), **Silvia Caporotundo** (Ospedale Bassini, Cinisello Balsamo/MI), **Federica Cappuccia** (AORN Ospedale dei Colli, Napoli), **Tatiana Caputo** (Fondazione Onlus Casa di Riposo Città di Chiavenna/SO), **Paolo Caraceni** (Policlinico di Sant'Orsola - IRCCS Azienda Ospedaliero-Universitaria di Bologna), **Laura Carassale** (Ospedale Bassini, Cinisello Balsamo/MI), **Marco Cardinali** (Ospedale San Salvatore, Pesaro), **Giulia Carignano** (Azienda Ospedaliera Sante Croce e Carle, Cuneo), **Massimiliano Carlin** (Ospedale Cardinal Massaia, ASL di Asti), **Denis Carlini** (CRA Francesco Roncati - Domus Assistenza, Spilamberto/MO), **Silvia Carlini** (Ospedale Franchini, Santarcangelo di Romagna/RN), **Alessandro Carrafiello** (AORN San Giuseppe Moscati, Avellino), **Stefania Cartesio** (Fondazione Policlinico Universitario Agostino Gemelli IRCCS, Roma), **Anna Casanova** (Ospedale Santa Chiara, Trento), **Antonio Casella** (Residenza Protetta Chiara Luce, Casarza Ligure/GE), **Amalia Rita Caspanello** (Ospedale Cardinal Massaia, ASL di Asti), **Giuseppe Castiglia** (Ospedale Maggiore, Trieste), **Felice Catalano** (Ospedale Santa Maria della Misericordia, Rovigo), **Chiara Catellani** (Ospedale S. Anna, Castelnovo ne' Monti/RE), **Caterina Cattel** (Italian Hospital Group - Gruppo Korian, Roma), **Francesco Cavaleri** (Ospedale Fatebenefratelli, Milano), **Stefano Cavallero** (Ospedale Cardinal Massaia, ASL di Asti), **Andrea Cavalli** (Azienda Ospedaliera San Giovanni Addolorata, Roma), **Roberta Cavallo** (Ospedale Civile di Cuorgnè/TO), **Alessandro Cavarape** (Ospedale Santa Maria della Misericordia, Udine), **Sara Cazzavillan** (Ospedale Santa Maria della Misericordia, Rovigo), **Marco Cecchettani** (Presidio Ospedaliero Beata Vergine Consolata Fatebenefratelli, San Maurizio Canavese/TO), **Dania Cecchi** (RSA Coteto, Livorno), **Silvia Cecci** ( Ospedale San Valentino, Montebelluna/TV), **Moira Ceci** (Azienda Ospedaliera San Giovanni Addolorata, Roma), **Chiara Celentano** (Residenza Richelmy, Torino), **Paola Cena** (Azienda Ospedaliera Sante Croce e Carle, Cuneo), **Benedetta Cerasoli** (Istituto Geriatrico Camillo Golgi - ASP Golgi Redaelli, Abbiategrasso/MI), **Francesco Maria Ceravolo** (Casa Protetta San Domenico, Palermiti/CZ - RSA Santa Maria del Monte, Petrizzi/CZ), **Elisa Ceriani** (Ospedale Luigi Sacco, Milano), **Giovanna Cervati** (Ospedale Santa Maria della Misericordia, Rovigo), **Elena Cescutti** (Presidio Ospedaliero di San Daniele/UD), **Maria Valeria Cherchi** (Ospedale Sirai, Carbonia/SU), **Donatella Chessa** (Azienda Ospedaliero Universitaria di Sassari), **Antonino Chiarello** (Istituto Ospitale Magri Onlus, Urgnano/BG), **Valentina Chiusano** (Ospedale Cardinal Massaia, ASL di Asti), **Saverio Antonio Ciampi** (Ospedale San Giuseppe, Copertino/LE), **Sebastiano Cicco** (Ospedale Giovanni XXIII - Policlinico di Bari), **Luana Cimaduomo** (Azienda Ospedaliero-Universitaria Policlinico di Foggia), **Maria Luigia Cipollini** (Policlinico di Sant'Orsola - IRCCS Azienda Ospedaliero-Universitaria di Bologna), **Giovanna Cipriani** (Centro Servizi alla Persona Domenico Sartor, Castelfranco Veneto/TV), **Antonella Cipriani** (Fondazione Istituto Geriatrico La Pelucca Onlus, Sesto San Giovanni/MI), **Lucia Ciucciarelli** (Azienda Ospedaliero Universitaria Careggi, Firenze), **Giorgio Civitarese** (Fondazione IRCCS Policlinico San Matteo, Pavia), **Francesca Coccina** (Ospedale Civile Spirito Santo, Pescara), **Angelo Cocuzza** (Ospedale Santa Maria della Misericordia, Rovigo), **Chiara Cogliati** (Ospedale Luigi Sacco, Milano), **Martina Collareda** (Centro Servizi Sociali Villa Serena - Residenza Fontanelle di Trissino/VI, Residenza Giardino di Recoaro Terme/VI, Villa Serena di Valdagno/VI), **Elena Colli** (Ospedale Civico di Chivasso/TO), **Giovanna Colombo** (Ospedale di Circolo, Varese), **Alessio Comitangelo** (Ospedale Centrale Santissima Annunziata, Taranto), **Massimo Confalonieri** (Casa Di Riposo Giuseppe Gasparini, Rivergaro/PC), **Giulia Cono** (Ospedale Fondazione Poliambulanza, Brescia), **Francesca Conte** (IRCCS Ospedale San Raffaele, Milano), **Giuseppina Contri** (CRA Montese - Domus Assistenza, Montese/MO), **Eleonora Coppo** (Ospedale Cardinal Massaia, ASL di Asti), **Antonella Cordero** (Ospedale Civile di Saluzzo/CN), **Maria Grazia Corrado** (CRA Francesco Roncati - Domus Assistenza, Spilamberto/MO), **Fabrizio Corsini** (Ospedale Civile di Saluzzo/CN), **Giulia Corvalli** (Policlinico di Sant'Orsola - IRCCS Azienda Ospedaliero-Universitaria di Bologna), **Francesca Corzato** (Azienda Ospedaliera Universitaria Integrata di Verona), **Anna Costacurta** (Ospedale San Bassiano, Bassano del Grappa/VI), **Marianna Costarella** (Ospedale Israelitico, Roma), **Giulia Cozza** (Ospedale San Bassiano, Bassano del Grappa/VI), **Stefania Crivellari** (Ospedale Michele e Pietro Ferrero, Verduno/CN), **Eleonora Cucini** (Fondazione IRCCS San Gerardo dei Tintori, Monza), **Mario Antonio Cucumo** (Fondazione Elisabetta Germani Onlus, Cingia dè Botti/CR), **Rosa Curcio** (Ospedale Centrale Santissima Annunziata, Taranto), **Madalina Curea** (Gruppo Domus Assistenza - CRA Fili d'Argento, Pavullo nel Frignano/MO - CRA Francesco e Chiara, Pavullo nel Frignano/MO - CRA Montese, Montese/MO), **Chiara Cutaia** (Istituto Geriatrico Camillo Golgi - ASP Golgi Redaelli, Abbiategrasso/MI), **Mattia Da Rio** (Presidio Ospedaliero di San Daniele/UD), **Pierluigi Dal Santo** (Ospedale Santa Maria della Misericordia, Rovigo), **Cristina Dalle Carbonare** (Ospedale San Bassiano, Bassano del Grappa/VI), **Sarah Damanti** (IRCCS Ospedale San Raffaele, Milano), **Gennaro D'Angelo** (Azienda Ospedaliero Universitaria Pisana, Pisa), **Antonietta Daniele** (Casa Protetta San Domenico, Palermiti/CZ), **Annarita D'Auria** (Azienda Ospedaliero-Universitaria di Ferrara), **Federica Davolio** (Gruppo Domus Assistenza - CRA Alta Intensità Assistenziale, Castelfranco Emilia/MO - CRA Carlo Alberto Dalla Chiesa, Ravarino/MO - CRA Fili d'Argento, Pavullo nel Frignano/MO - CRA Francesco e Chiara, Pavullo nel Frignano/MO - CRA Francesco Roncati, Spilamberto/MO - CRA Il Carpine, Carpi/MO - CRA Il Quadrifoglio, Carpi/MO - CRA Montese, Montese/MO - CRA Sandro Pertini, Soliera/MO - CRA Vittoria ed Ermanno Gorrieri, Modena), **Stefano De Carli** (Presidio Ospedaliero di San Daniele/UD), **Carlo De Cesare** (Fondazione Istituto Geriatrico La Pelucca Onlus, Sesto San Giovanni/MI), **Paolo De Colle** (Ospedale Maggiore, Trieste), **Tiziana De Gregorio** (Ospedale San Giovanni di Dio e Sant'Isidoro, Giarre/CT), **Massimo De Martinis** (Ospedale Civile Maria SS. dello Splendore, Giulianova/TE), **Laura De Niederhausern** (CRA Carlo Alberto Dalla Chiesa - Domus Assistenza, Ravarino/MO), **Angela De Rossi** (RSA Relaxxi Santa Maria dei Battuti, Noale/VE), **Stefano De Santis** (Azienda Ospedaliera San Camillo Forlanini, Roma), **Antonio De Simone** (RSA La Quiete, Castiglione Cosentino/CS), **Chiara De Simone** (CRA Sandro Pertini - Domus Assistenza, Soliera/MO), **Angela De Solis** (Ospedale Civile Maria SS. dello Splendore, Giulianova/TE), **Davide De Vito** (Ospedale Civico di Chivasso/TO), **Barbara Defrancisco** (RSA Alchemica, Torino), **Giulia Deias** (Fondazione Policlinico Universitario Agostino Gemelli IRCCS, Roma), **Elena Del Giudice** (Azienda Ospedaliera San Giovanni Addolorata, Roma), **Ilaria Del Lungo** (Ospedale San Giovanni di Dio, Firenze), **Raffaella Dell'Oro** (Fondazione IRCCS San Gerardo dei Tintori, Monza), **Giorgia Demelas** (Ospedale Santissima Trinità, Cagliari), **Antonio D'Errico** (Ospedale Misericordia, Grosseto), **Giovambattista Desideri** (Azienda Ospedaliero-Universitaria Policlinico Umberto I, Roma), **Sara Di Bartolomeo** (Ospedale San Salvatore, L'Aquila), **Tiziana Di Cocco** (RSA Coteto, Livorno), **Manuela Di Fabio** (CRA I Lecci, Parma), **Vincenzo Di Francesco** (Azienda Ospedaliera Universitaria Integrata di Verona), **Giuseppe Di Gioia** (Azienda Ospedaliero-Universitaria Policlinico di Foggia), **Fabiana Di Girolamo** (Ospedale G. B. Grassi, Roma), **Monica Di Lanzo** (Ospedale Civile Spirito Santo, Pescara), **Debora Di Pasquale** (RSA Salus, Roma), **Michela Di Pol** (Centro Servizi Ca' dei Fiori, Quarto d’Altino/VE), **Antonio Di Sabatino** (Fondazione IRCCS Policlinico San Matteo, Pavia), **Marco Domenicali** (Ospedale Santa Maria delle Croci, Ravenna), **Marco Paolo Donadini** (Ospedale di Circolo, Varese), **Teresa Donadio** (RSA Villa Azzurra, Roseto Capo Spulico/CS), **Francesco Donvito** (Ospedale Generale Regionale F. Miulli, Acquaviva delle Fonti/BA), **Florida Driza** (CRA di Vignola - ASP Terre di Castelli, Vignola/MO), **Elisa Durante** (Fondazione Policlinico Universitario Agostino Gemelli IRCCS, Roma), **Margherita Dusi** (Azienda Socio Sanitaria Territoriale degli Spedali Civili di Brescia), **Luca Dutto** (Ospedale Civile di Saluzzo/CN), **Daniele Elmi** (Casa di Cura Villa Immacolata, Viterbo), **Giacomo Emmi** (Ospedale di Cattinara, Trieste), **Raffaele Esposito** (CRA Le Tamerici, Parma), **Amedeo Evangelisti** (Ospedale Civile A. Costa, Porretta Terme/BO), **Alessia Fabbri** (Azienda Ospedaliero Universitaria Careggi, Firenze), **Dario Falcone** (Ospedale del Delta, Lagosanto/FE), **Stefano Fascetti** (Ospedale della Versilia, Lido di Camaiore/LU), **Lucia Fasciani** (Ospedale Civile Spirito Santo, Pescara), **Cristina Faustini** (Ospedale Civile di Iseo/BS), **Chiara Fazio** (Ospedale della Val d'Arda, Fiorenzuola d’Arda/PC) **Silvia Daniela Umbertina Fedeli** (Ospedale S. Anna, Castelnovo ne' Monti/RE), **Marika Feroldi** (Azienda Socio Sanitaria Territoriale degli Spedali Civili di Brescia), **Annalisa Ferrante** (Azienda Ospedaliera San Giovanni Addolorata, Roma), **Maria Cristina Ferrara** (Fondazione IRCCS San Gerardo dei Tintori, Monza), **Sara Ferrarini** (Ospedale San Carlo Borromeo, Milano), **Cinzia Ferreri** (Ospedale Civile di Saluzzo/CN), **Fortunato Ferrero** (Fondazione Istituto Geriatrico La Pelucca Onlus, Sesto San Giovanni/MI), **Christian Ferro** (RSA di Sant'Angelo di Brolo/ME), **Mariano Festa** (Azienda Ospedaliero Universitaria San Giovanni di Dio Ruggi d'Aragona, Salerno), **Pamela Festa** (Residenza al Castello, Alpignano/TO), **Irene Filipetto** (Casa di Cura Sileno e Anna Rizzola, San Donà di Piave/VE), **Chiara Filippini** (Fondazione Casa di Industria Onlus, Brescia), **Francesca Fiorentino** (Azienda Ospedaliero Universitaria Careggi, Firenze), **Alberto Fisichella** (Azienda Ospedaliero-Universitaria di Parma), **Giorgia Fontana** (Azienda Ospedaliera Universitaria Integrata di Verona), **Marino Formilan** (Residenza Riviera del Brenta, Dolo/VE), **Chiara Fornasier** (Ospedale San Valentino, Montebelluna/TV), **Paolo Forzan** (RSA AltaVita IRA, Padova), **Giampiero Frasca** (Fondazione IRCCS San Gerardo dei Tintori, Monza), **Elisabetta Fumo** (Presidio Ospedaliero di San Daniele/UD), **Antonio Fusari** (Fondazione Istituto Carlo Vismara - Giovanni De Petri Onlus, San Bassano/CR), **Giulia Fusaro** (Ospedale di Cattinara, Trieste), **Giulia-Francesca Fusi** (Ospedale San Leopoldo Mandic, Merate/LC), **Umberto Giuseppe Galasso** (Azienda Ospedaliera San Giovanni Addolorata, Roma), **Caterina Galetti** (Policlinico di Sant'Orsola - IRCCS Azienda Ospedaliero-Universitaria di Bologna), **Paola Galetti** (Fondazione Istituto dei Ciechi di Milano), **Elisabetta Galli** (CRA Carlo Alberto Dalla Chiesa - Domus Assistenza, Ravarino/MO), **Roberto Galliano** (Azienda Ospedaliera Sante Croce e Carle, Cuneo), **Giulia Gallotta** (Ospedale Santa Maria delle Stelle, Melzo/MI), **Cristiana Galuppi** (Ospedale Santa Maria della Misericordia, Rovigo), **Giovanna Gambino** (Ospedale San Carlo Borromeo, Milano), **Federica Gandini** (Fondazione Istituto Geriatrico La Pelucca Onlus, Sesto San Giovanni/MI), **Nadia Gandolfi** (CRA Alta Intensità Assistenziale - Domus Assistenza, Castelfranco Emilia/MO), **Andrea Ganz** (Ospedale San Valentino, Montebelluna/TV), **Debora Gari** (Centro Servizi Contarini, Mestre/VE), **Antonio Garo** (RSA San Domenico, Lamezia Terme/CZ - RSA Villa Elisabetta, Cortale/CZ), **Maria Garro** (Azienda Ospedaliera Sante Croce e Carle, Cuneo), **Lorenza Gaspari** (Ospedale Civile Spirito Santo, Pescara), **Chiara Gasperoni** (Istituto per la Sicurezza Sociale, San Marino/RSM), **Monica Geminiani** (Ospedale del Delta, Lagosanto/FE), **Valentina Gemo** (Ospedale degli Infermi, Faenza/RA), **Bruno Gerardo** (Ospedale Regina Montis Regalis, Mondovì/CN), **Nives Ghezzi** (Ospedale Villa Igea, Modena), **Lorenzo Ghiadoni** (Azienda Ospedaliero Universitaria Pisana, Pisa), **Maria Francesca Ghiara** (RSA Cottolengo, Fornacette/PI), **Loredana Ghidoni** (Fondazione Elisabetta Germani Onlus, Cingia dè Botti/CR), **Giuseppina Giachetta** (Ospedale Civile Spirito Santo, Pescara), **Luana Giacobbe** (Ospedale Santa Maria della Misericordia, Rovigo), **Benedetta Giacomelli** (Azienda Ospedaliero Universitaria Pisana, Pisa), **Giulia Alessandra Gianoglio** (Ospedale Cardinal Massaia, ASL di Asti), **Giordano Gianotti** (Ospedale Simiani, Loiano/BO), **Giulia Giardini** (RSA di Cascia/PG), **Marianna Gigliotti De Fazio** (Azienda Ospedaliera Universitaria Gaetano Martino, Messina), **Fabio Gilioli** (Ospedale Santa Maria Bianca, Mirandola/MO), **Stefania Giordano** (RSA Salus, Roma), **Silvio Giorgi** (Istituto Geriatrico Camillo Golgi - ASP Golgi Redaelli, Abbiategrasso/MI), **Desireé Giudice** (ARNAS Ospedale Garibaldi-Nesima, Catania), **Marco Giuliani** (RSA Le Grazie, Terni), **Alice Giuliodori** (Residenza Posatora - Gruppo Zaffiro, Ancona), **Andrea** **Giusti** (Ospedale Alessandro Manzoni, Lecco), **Paola Gnerre** (Ospedale San Paolo, Savona), **Claudia Gobbi** (Ospedale Civile di Cuorgnè/TO), **Francesca Greppi** (Ospedale Cardinal Massaia, ASL di Asti), **Yasseni Griffith** (Ospedale Santa Maria della Misericordia, Udine), **Cristina Grilli** (Casa Protetta Villa Giulia, Bologna), **William Grimaldi** (Ospedale Alessandro Manzoni, Lecco), **Mario Guarino** (AORN Ospedale dei Colli, Napoli), **Gianbattista Guerrini** (RSA Arici Sega - Fondazione Brescia Solidale, Brescia), **Alberto Guida** (CRA Le Tamerici, Parma), **Karine Guidi** (Istituto per la Sicurezza Sociale, San Marino/RSM), **Federica Gullotto** (ARNAS Ospedale Garibaldi-Nesima, Catania), **Sophia Hassani** (Presidio Ospedaliero di Palmanova/UD), **Guido Horn** (Fondazione Policlinico Universitario Agostino Gemelli IRCCS, Roma), **Giuseppe Ielo** (Ospedale della Val d'Arda, Fiorenzuola d’Arda/PC), **Egidio Imbalzano** (Azienda Ospedaliera Universitaria Gaetano Martino, Messina), **Alessandra Imoscopi** (Ospedale Santa Maria della Misericordia, Rovigo), **Nicola Innocente** (Ospedale Santa Maria della Misericordia, Udine), **Mariapia Iovenitti** (Ospedale San Salvatore, L'Aquila), **Valerio Alex Ippolito** (RSA La Quiete, Castiglione Cosentino/CS - RSA Villa Azzurra, Roseto Capo Spulico/CS), **Alexandra Istoc** (IRCCS Istituto Oncologico di Candiolo/TO), **Angela Iurlaro** (Fondazione Policlinico Universitario Agostino Gemelli IRCCS, Roma), **David Kanah** (Istituto Geriatrico Camillo Golgi - ASP Golgi Redaelli, Abbiategrasso/MI), **Bjanka Krinulovic** (Centro Servizi Sociali Villa Serena - Residenza Fontanelle di Trissino/VI, Residenza Giardino di Recoaro Terme/VI, Villa Serena di Valdagno/VI), **Alfio La Spina** (ARNAS Ospedale Garibaldi-Nesima, Catania), **Francesco Landi** (Fondazione Policlinico Universitario Agostino Gemelli IRCCS, Roma), **Valentina Lanzilotti** (Presidio Ospedaliero di San Daniele/UD), **Federico Lari** (Ospedale di Budrio/BO), **Angela Lazzari** (Centro Servizi Ca' dei Fiori, Quarto d’Altino/VE), **Alessandro Lazzarin** (Ospedale Santa Maria della Misericordia, Rovigo), **Maria Lazzaroni** (Casa Di Riposo Giuseppe Gasparini, Rivergaro/PC), **Daniele Le Serre** (Ospedale Cardinal Massaia, ASL di Asti), **Elisa Lenotti** (Fondazione Elisabetta Germani Onlus, Cingia dè Botti/CR), **Marco Vincenzo Lenti** (Fondazione IRCCS Policlinico San Matteo, Pavia), **Salvatore Lenti** (Ospedale Lorenzo Bonomo, Andria/BT), **Debora Leo** (CRA Sandro Pertini - Domus Assistenza, Soliera/MO), **Francesca Leonardi** (Ospedale di Sassuolo/MO), **Dario Leone** (IRCCS Istituto Oncologico di Candiolo/TO), **Simona Leoni** (Ospedale di Bentivoglio/BO), **Monica Lera** **Marchetti** (RSA Casa Amica, Fossato Serralta/CZ), **Claudio Letizia** (Azienda Ospedaliero-Universitaria Policlinico Umberto I, Roma), **Stefania Levi** (ARNAS Ospedale Garibaldi-Nesima, Catania), **Mattia Lillu** (ARNAS Ospedale G. Brotzu, Cagliari), **Carlo Longato** (Azienda Ulss 3, Venezia)**, Elena Longo** (IRCCS Ospedale Policlinico San Martino, Genova), **Giancarlo Lorenzato** (Centro Servizi Anziani Tintoretto, Pianiga/VE), **Ilaria Lospinuso** (Azienda Ospedaliero-Universitaria Policlinico Umberto I, Roma), **Giulia Lucarelli** (Ospedale San Jacopo, Pistoia), **Luca Lugli** (CRA Il Quadrifoglio - Domus Assistenza, Carpi/MO), **Ester Lupinacci** (RSA San Domenico, Lamezia Terme/CZ), **Myriam Macaluso** (Fondazione Policlinico Universitario Agostino Gemelli IRCCS, Roma), **Maria Macchiarulo** (Policlinico di Sant'Orsola - IRCCS Azienda Ospedaliero-Universitaria di Bologna), **Paolo Maggi** (Azienda Speciale Cremona Solidale, Cremona), **Dario Maggioni** (Ospedale Sant'Anna, Como), **Caterina Magliocchetti** (Residenza Protetta San Sebastiano, Panicale/PG), **Sara Maineri** (Centro Servizi alla Persona Domenico Sartor, Castelfranco Veneto/TV), **Federica Maino** (Ospedale Sant'Anna, Como), **Alba Malara** (Fondazione ANASTE Humanitas, Roma - Casa Protetta Madonna del Rosario, Lamezia Terme/CZ - Casa Protetta Villa Santo Stefano, Santo Stefano di Rogliano/CS - RSA San Domenico, Lamezia Terme/CZ - RSA Villa Elisabetta, Cortale/CZ), **Emma Manassero** (Centro Servizi per Anziani Nazaret, Zelarino/VE), **Carla Manca** (Opere Pie Riunite Devoto Marini Sivori, Lavagna/GE), **Antonella Mandas** (Azienda Ospedaliero-Universitaria di Cagliari - Azienda Socio-sanitaria Locale di Cagliari), **Giuseppe Mandraffino** (Azienda Ospedaliera Universitaria Gaetano Martino, Messina), **Tania Manfredini** (CRA Carlo Alberto Dalla Chiesa - Domus Assistenza, Ravarino/MO), **Elisabetta Mangili** (Fondazione IRCCS San Gerardo dei Tintori, Monza), **Pietro Mansi** (Azienda Ospedaliera Universitaria Federico II, Napoli), **Mauro Mantega** (Ospedale Sirai, Carbonia/SU), **Stefano Mantovani** (RSA Don Giuseppe Cuni, Magenta/MI), **Marco Marabotto** (Azienda Ospedaliera Sante Croce e Carle, Cuneo), **Elisabetta Marcon** (Ospedale San Valentino, Montebelluna/TV), **Alessandra Marengoni** (Presidio Ospedaliero di Montichiari/BS), **Andrea Maria Maresca** (Ospedale Sant'Anna, Como), **Gerarda Maresca** (Azienda Ospedaliero Universitaria San Giovanni di Dio Ruggi d'Aragona, Salerno), **Sara Marin** (Ospedale San Bassiano, Bassano del Grappa/VI), **Sara Marinelli** (Policlinico di Sant'Orsola - IRCCS Azienda Ospedaliero-Universitaria di Bologna), **Valentina Maritato** (RSA Villa Torano, Torano Castello/CS), **Mariaelena Marolla** (Ospedali Riuniti Padova Sud, Monselice/PD), **Alessandro Marongiu** (Ospedale Santissima Trinità, Cagliari), **Pietro Martella** (Ospedale Villa Igea, Modena), **Lucia Martinelli** (Ospedale San Valentino, Montebelluna/TV), **Laura Martinoli** (Ospedale Uboldo, Cernusco sul Naviglio/MI), **Erica Masini** (CRA Il Carpine - Domus Assistenza, Carpi/MO), **Annalisa Mastrapasqua** (Azienda Ospedaliera Sante Croce e Carle, Cuneo), **Franco Mastroianni** (Ospedale Generale Regionale F. Miulli, Acquaviva delle Fonti/BA), **Luca Matteucci** (Ospedale San Carlo Borromeo, Milano), **Massimo Mattioli** (Ospedale San Salvatore, Pesaro), **Michela Mattioli** (RSA di Cascia/PG), **Sabrina Mauri** (Villa San Benedetto Menni, Albese con Cassano/CO), **Roberta Medici** (CRA Vittoria ed Ermanno Gorrieri - Domus Assistenza, Modena), **Graziella Megna** (Casa Protetta Madonna del Rosario, Lamezia Terme/CZ), **Remo Melchio** (Azienda Ospedaliera Sante Croce e Carle, Cuneo), **Eleonora Meloni** (Fondazione Policlinico Universitario Agostino Gemelli IRCCS, Roma), **Giulia Menardi** (Azienda Ospedaliero-Universitaria Città della Salute e della Scienza di Torino), **Rita Mennella** (CRA Francesco e Chiara - Domus Assistenza, Pavullo nel Frignano/MO), **Elena Migliore** (Ospedale Santissima Annunziata, Savigliano/CN), **Marina Migliori** (Policlinico di Sant'Orsola - IRCCS Azienda Ospedaliero-Universitaria di Bologna), **Alessandra Minnucci** (Ospedale di Sassuolo/MO), **Alberto Moggi Pignone** (Azienda Ospedaliero Universitaria Careggi, Firenze), **Gabriele Mogliarisi** (IRCCS Ospedale San Raffaele, Milano), **Fiammetta Monacelli** (IRCCS Ospedale Policlinico San Martino, Genova), **Cinzia Monaco** (IRCCS Ospedale Sacro Cuore Don Calabria, Negrar/VR), **Federica Moniale** (Centro Servizi San Giobbe, Venezia), **Hanane Monir** (CRA Vittoria ed Ermanno Gorrieri - Domus Assistenza, Modena), **Andrea Montagnani** (Ospedale Misericordia, Grosseto), **Luca Montanari** (Opere Pie Riunite Devoto Marini Sivori, Lavagna/GE), **Lidia Montemurro** (ASST Grande Ospedale Metropolitano Niguarda, Milano), **Giampiero Monti** (Ospedale San Carlo Borromeo, Milano), **Riccardo Monti** (Ospedale Cardinal Massaia, ASL di Asti), **Fabio Monzani** (RSA Cottolengo, Fornacette/PI), **Carmela Morace** (Azienda Ospedaliera Universitaria Gaetano Martino, Messina), **Alessandro Morandi** (Azienda Speciale Cremona Solidale, Cremona), **Camilla Morelli** (Azienda Socio Sanitaria Territoriale degli Spedali Civili di Brescia), **Mirna Morì** (RSA Coteto, Livorno), **Chiara Morichetti** (Ospedale Sant'Anna, Como), **Luca Morolli** (Ospedale Franchini, Santarcangelo di Romagna/RN), **Federica Moscucci** (Azienda Ospedaliero-Universitaria Policlinico Umberto I, Roma), **Susanna Motta** (Ospedale di Carate Brianza/MB), **Maria Lorenza Muiesan** (Azienda Socio Sanitaria Territoriale degli Spedali Civili di Brescia), **Luigi Muraca** (RSA Villa Azzurra, Roseto Capo Spulico/CS), **Chiara Mussi** (Ospedale Civile Sant'Agostino Estense, Baggiovara/MO), **Andrea Musu** (CRA IX Gennaio, Modena), **Francesca Musumeci** (Ospedale di Sassuolo/MO), **Luca Naldi** (Azienda Ospedaliero Universitaria Careggi, Firenze), **Marzia Napoleoni** (Azienda Ospedaliera San Camillo Forlanini, Roma), **Francesca Negretti** (RSA Arici Sega - Fondazione Brescia Solidale, Brescia), **Asimeko Nexhi** (CRA Fili d'Argento - Domus Assistenza, Pavullo nel Frignano/MO), **Filippo Niccolai** (Azienda Ospedaliero Universitaria Pisana, Pisa), **Laura Norelli** (Ospedale Cardinal Massaia, ASL di Asti), **Serena Notarstefano** (Ospedale Uboldo, Cernusco sul Naviglio/MI), **Maria Elena Novielli** (Ospedale Generale Regionale F. Miulli, Acquaviva delle Fonti/BA), **Vanessa Nunziata** (Ospedale Infermi, Rimini), **Valerio Oliva** (Ospedale Centrale Santissima Annunziata, Taranto), **Graziano Onder** (Fondazione Policlinico Universitario Agostino Gemelli IRCCS, Roma), **Pamela Orrù** (Ospedale Civile Maria SS. dello Splendore, Giulianova/TE), **Patrizia Pagliari** (RSA Villa San Clemente, Villasanta/MB), **Stefania Pagnotta** (Azienda Ospedaliera San Giovanni Addolorata, Roma), **Fiammetta Pagnozzi** (Ospedale Civico di Chivasso/TO), **Antonella Palimodde** (Ospedale Santissima Trinità, Cagliari), **Francesca Pallavicino** (Ospedale Michele e Pietro Ferrero, Verduno/CN), **Lorenzo Palleschi** (Azienda Ospedaliera San Giovanni Addolorata, Roma), **Francesco Palmese** (Ospedale Santa Maria delle Croci, Ravenna), **Vincenzo Ostilio Palmieri** (Ospedale Giovanni XXIII - Policlinico di Bari), **Monica Paltro** (Ospedale Cardinal Massaia, ASL di Asti), **Maria Valentina Pamio** (Ospedale San Bassiano, Bassano del Grappa/VI), **Riccardo Bogdan Pana** (Ospedale G.B. Morgagni - L. Pierantoni, Forlì), **Luigi Pansini** (RSA Villa Torano, Torano Castello/CS), **Filomena Panzone** (Ospedale San Martino, Oristano), **Samantha Paruzzolo** (Ospedale San Valentino, Montebelluna/TV), **Michela Passamonte** (Ospedale di Chiavenna/SO), **Lorenzo Passarini** (Fondazione Angelo Passerini Onlus, Nozza di Vestone/BS), **Carlo Alberto Pastura** (Azienda Ospedaliero Universitaria Renato Dulbecco, Catanzaro), **Monica Patrignani** (Ospedale Santa Maria delle Croci, Ravenna), **Martina Pazzaglia** (Ospedale Misericordia, Grosseto), **Enrica Pea** (Azienda Ospedaliero Universitaria Pisana, Pisa), **Maria Tecla Pecci** (Azienda Ospedaliero-Universitaria Policlinico Umberto I, Roma), **Claudio Pedone** (Fondazione Policlinico Universitario Campus Bio-Medico, Roma), **Milena Pedroni** (Fondazione Onlus Casa di Riposo Città di Chiavenna/SO), **Laura Pelanconi** (Fondazione Onlus Casa di Riposo Città di Chiavenna/SO), **Anna Pellenghi** (Azienda Socio Sanitaria Territoriale degli Spedali Civili di Brescia), **Francesca Pellicciotti** (Arcispedale Santa Maria Nuova, Reggio Emilia), **Giampaolo Peracchiotti** (RSA Alchemica, Torino), **Laura Perale** (Presidio Ospedaliero di Palmanova/UD), **Daniela Perin** (Centro Servizi Anziani Tintoretto, Pianiga/VE), **Valeria Perini** (Centro Servizi Anziani Felice Federico Casson, Chioggia/VE), **Pier Francesco Perna** (Casa Protetta Villa Santo Stefano, Santo Stefano di Rogliano/CS), **Luigi Maria Pernigotti** (Residenza al Castello, Alpignano/TO), **Agostino Perri** (RSA La Quiete, Castiglione Cosentino/CS), **Eleonora Persolja** (Ospedale Santa Maria della Misericordia, Udine), **Francesco Perticone** (RSA San Francesco Hospital, Settingiano/CZ), **Chiara Pes** (Azienda Ospedaliero Universitaria di Sassari), **Maria Valeria Pes** (Residenza Anziani Adele Zara, Mira/VE), **Nunzio Petrolini** (Ospedale di Sassuolo/MO), **Florinda Petruzzellis** (Ospedale Santa Maria della Misericordia, Rovigo), **Laura Piantoni** (RSA Madre Teresa di Calcutta, Brescia), **Ilaria Pietropaolo** (Ospedale Cardinal Massaia, ASL di Asti), **Gloria Pina** (Villa San Benedetto Menni, Albese con Cassano/CO), **Elena Pinardi** (Fondazione IRCCS San Gerardo dei Tintori, Monza), **Elisa Pinna** (Ospedale Santissima Trinità, Cagliari), **Nicola Pirisi** (Ospedale Sirai, Carbonia/SU), **Martina Pirola** (Ospedale Fatebenefratelli, Milano), **Anna Italia Pisacreta** (Ospedale San Rocco, Sessa Aurunca/CE), **Erika Pistone** (Ospedale Civile di Saluzzo/CN), **Gaia Pistoresi** (Centro Servizi Anziani Tintoretto, Pianiga/VE), **Luciano Pittau** (Ospedale Santissima Trinità, Cagliari), **Francesca Vera Pittella** (Istituto Geriatrico Piero Redaelli - ASP Golgi Redaelli, Milano9, **Elisa Tunde Podolyak** (Casa Protetta Madonna del Rosario, Lamezia Terme/CZ), **Marta Poggi** (Ospedale Bassini, Cinisello Balsamo/MI), **Enrica Polacco** (Ospedale G. B. Grassi, Roma), **Francesca Poli** (Azienda Ospedaliero Universitaria Pisana, Pisa), **Fulvio Pomero** (Ospedale Michele e Pietro Ferrero, Verduno/CN), **Maria Ponzetto** (Presidio Ospedaliero Beata Vergine Consolata Fatebenefratelli, San Maurizio Canavese/TO ), **Valentina Popescu Janu** (Ospedale Luigi Sacco, Milano), **Erasmo Porfido** (Ospedale Centrale Santissima Annunziata, Taranto), **Letizia Pozzebon** (Ospedale San Valentino, Montebelluna/TV), **Elisa Pozzobon** (Centro Servizi San Giobbe, Venezia), **Giorgia Prampolini** (Ospedale S. Anna, Castelnovo ne' Monti/RE), **Marianna Libera Prencipe** (Ospedale Giovanni XXIII - Policlinico di Bari), **Roberto Presta** (Azienda Ospedaliero-Universitaria Città della Salute e della Scienza di Torino), **Anna Chiara Prezioso** (CRA di Vignola - ASP Terre di Castelli, Vignola/MO), **Ilenia Prioletta** (RSA Le Grazie, Terni), **Marco Proietti** (IRCCS Istituti Clinici Scientifici Maugeri, Milano), **Porzia Prudente** (Ospedale San Carlo Borromeo, Milano), **Rosanna Pullia** (Casa Protetta Madonna del Rosario, Lamezia Terme/CZ - RSA San Domenico, Lamezia Terme/CZ - RSA Villa Elisabetta, Cortale/CZ), **Valeria Quaranta** (Azienda Ospedaliera Sante Croce e Carle, Cuneo), **Davide Quattrociocchi** (Ospedale Santa Chiara, Trento), **Angela Raciti** (Ospedale Santa Maria Bianca, Mirandola/MO), **Fatima Angela Ramillano** (CRA Il Carpine - Domus Assistenza, Carpi/MO), **Silvio Raspo** (Azienda Ospedaliera Sante Croce e Carle, Cuneo), **Giuseppe Re** (Ospedale Centrale Santissima Annunziata, Taranto), **Francesca Regina** (Residenza Richelmy, Torino), **Francesca Remelli** (Azienda Ospedaliero-Universitaria di Ferrara), **Maurizio Renda** (Fondazione Istituto G. Giglio, Cefalù/PA), **Giuseppe Rengo** (Azienda Ospedaliera Universitaria Federico II, Napoli), **Tiziana Restivo** (Azienda Ospedaliero Universitaria Pisana, Pisa), **Giorgio Ricci** (RSA Villa San Clemente, Villasanta/MB), **Alice Riccò** (Ospedale di Carate Brianza/MB), **Cristian Riello** (RSA Relaxxi Santa Maria dei Battuti, Noale/VE), **Filippo Rinaldi** (Ospedale San Valentino, Montebelluna/TV), **Giada Rinaldi** (University of Florence), **Gianluca Rinaldi** (Azienda Ospedaliera Sante Croce e Carle, Cuneo), **Piero Riva** (Ospedale Cardinal Massaia, ASL di Asti), **Caterina Rizzo** (RSA La Quiete, Castiglione Cosentino/CS), **Valentina Romano** (Fondazione Casa di Industria Onlus, Brescia), **Giuliano Ronco** (Ospedale Cardinal Massaia, ASL di Asti), **Caterina Rontauroli** (Ospedale Civile Sant'Agostino Estense, Baggiovara/MO), **Vilma Roso** (Residenza Protetta Brancaleoni, Panicale/PG), **Arianna Rossi** (Ospedale Santa Croce, Moncalieri/TO), **Paolo Dionigi Rossi** (Ospedale San Leopoldo Mandic, Merate/LC), **Roberta Rossi** (RSA Don Giuseppe Cuni, Magenta/MI), **Sofia Rossini** (Residenza Cappuccini - Gruppo Zaffiro, Ancona), **Carlo Rostagno** (Azienda Ospedaliero Universitaria Careggi, Firenze), **Alessandra Rota** (Istituto Ospitale Magri Onlus, Urgnano/BG), **Patrizia Rovere-Querini** (IRCCS Ospedale San Raffaele, Milano), **Ezio Ruggeri** (RSA Pietro Beretta, Gardone Val Trompia/BS), **Francesca Ruggeri** (CRA Carlo Alberto Dalla Chiesa - Domus Assistenza, Ravarino/MO), **Federico Ruggiero** (AORN San Giuseppe Moscati, Avellino), **Stefano Ruggiero** (Ospedale G.B. Morgagni - L. Pierantoni, Forlì), **Silvana Ruli** (Istituto per la Sicurezza Sociale, San Marino/RSM), **Simone Sabatini** (RSA di Cascia/PG), **Alice Sabbadini** (Fondazione Policlinico Universitario Campus Bio-Medico, Roma), **Isabella Salimbeni** (Fondazione Elisabetta Germani Onlus, Cingia dè Botti/CR), **Francesco Salis** (Azienda Ospedaliero-Universitaria di Cagliari), **Elisa Salsano** (AORN San Giuseppe Moscati, Avellino), **Anna Salvatori** (RSA di Cascia/PG), **Chiara Sambalino** (Ospedale San Giuseppe, Empoli/FI), **Claudio Santini** (Ospedale G. B. Grassi, Roma), **Brunilda Saraci** (CRA Vittoria ed Ermanno Gorrieri - Domus Assistenza, Modena), **Giorgia Sasia** (Azienda Ospedaliera Sante Croce e Carle, Cuneo), **Carolina Sassi** (Ospedale Sant'Anna, Como), **Valeria Savona** (CRA Vittoria ed Ermanno Gorrieri - Domus Assistenza, Modena), **Matteo Sbattella** (ASST Grande Ospedale Metropolitano Niguarda, Milano), **Delia Scagliusi** (Ospedale Fracastoro, San Bonifacio/VR), **Luca Scaldaferri** (Casa di Cura Sileno e Anna Rizzola, San Donà di Piave/VE), **Riccardo Scaparrotti** (Ospedale G.B. Morgagni - L. Pierantoni, Forlì), **Elisa Schiavetta** (Ospedale San Paolo, Savona), **Francesca Schiavetti** (Ospedale G. B. Grassi, Roma), **Anna Maria Schimizzi** (Ospedale Santa Maria della Pietà, Camerino/MC), **Angela Sciacqua** (Azienda Ospedaliero Universitaria Renato Dulbecco, Catanzaro), **Giovanni Scomparin** (Casa di Cura Sileno e Anna Rizzola, San Donà di Piave/VE), **Raffaella Maria Scoyni** (Azienda Ospedaliera San Camillo Forlanini, Roma), **Rosa Scurti** (Ospedale Civile Spirito Santo, Pescara), **Marta Secci** (IRCCS Ospedale San Raffaele, Milano), **Leonardo Sechi** (Ospedale Santa Maria della Misericordia, Udine), **Piero Secreto** (Presidio Ospedaliero Beata Vergine Consolata Fatebenefratelli, San Maurizio Canavese/TO ), **Raffaella Selleri** (Fondazione Policlinico Universitario Campus Bio-Medico, Roma), **Simona Serafini** (Ospedale Cardinal Massaia, ASL di Asti), **Ivana Serino** (Azienda Ospedaliera Universitaria Federico II, Napoli), **Fabiana Serra** (Ospedale Franchini, Santarcangelo di Romagna/RN), **Adriana Debora Servello** (Azienda Ospedaliero-Universitaria Policlinico Umberto I, Roma), **Gaetano Serviddio** (Azienda Ospedaliero-Universitaria Policlinico di Foggia), **Mattia Seu** (Presidio Ospedaliero di San Daniele/UD), **Sara Simeone** (CRA Il Quadrifoglio - Domus Assistenza, Carpi/MO), **Luisa Sist** (Policlinico di Sant'Orsola - IRCCS Azienda Ospedaliero-Universitaria di Bologna), **Andrea Soccodato** (Ospedale Civile di Sondrio), **Marta Soddu** (Ospedale San Leopoldo Mandic, Merate/LC), **Costanza Sogno** (Ospedale Civile di Saluzzo/CN), **Stefania Soldi** (Fondazione Elisabetta Germani Onlus, Cingia dè Botti/CR), **Fausto Spadea** (RSA Casa Amica, Fossato Serralta/CZ), **Giovanni Squadrito** (Azienda Ospedaliera Universitaria Gaetano Martino, Messina), **Daniele Staffa** (Casa Protetta Villa Santo Stefano, Santo Stefano di Rogliano/CS), **Loredana Staglianò** (Ospedale San Giuseppe, Empoli/FI), **Carmen Stanciu** (Opere Pie Riunite Devoto Marini Sivori, Lavagna/GE), **Vanni Stangherlin** (Azienda Ulss 3, Venezia), **Alessia Stano** (CRA Il Quadrifoglio - Domus Assistenza, Carpi/MO), **Alessia Stingo** (Ospedale Santa Maria delle Croci, Ravenna), **Antonietta Storelli** (Azienda Ospedaliero-Universitaria di Parma), **Vincenza Suriano** (Ospedale Lorenzo Bonomo, Andria/BT), **Luca Tagliafico** (IRCCS Ospedale Policlinico San Martino, Genova), **Giovanni Talerico** (Azienda Ospedaliera San Giovanni Addolorata, Roma), **Rosa Talerico** (Fondazione Policlinico Universitario Agostino Gemelli IRCCS, Roma), **Teresa Talerico** (Ospedale Civile A. Costa, Porretta Terme/BO), **Roberto Tarquini** (Ospedale San Giuseppe, Empoli/FI), **Andrea Tedde** (Azienda Ospedaliero Universitaria di Sassari), **Greta Tedesco** (Policlinico di Sant'Orsola - IRCCS Azienda Ospedaliero-Universitaria di Bologna), **Elena Temponi** (RSA Pietro Beretta, Gardone Val Trompia/BS), **Fabiana Tezza** (Ospedali Riuniti Padova Sud, Monselice/PD), **Vittoria Tibaldi** (Azienda Ospedaliera Sante Croce e Carle, Cuneo), **Daniela Tirotta** (Ospedale G.B. Morgagni - L. Pierantoni, Forlì), **Giulia Tiscione** (Ospedale della Versilia, Lido di Camaiore/LU), **Moreno Tomaello** (Centro Servizi per Anziani Nazaret, Zelarino/VE), **Sara Tonini** (Ospedale Civile di Saluzzo/CN), **Singora Tonti** (Istituto per la Sicurezza Sociale, San Marino/RSM), **Monica Torrini** (Azienda Ospedaliero Universitaria Careggi, Firenze), **Lidia Tosi** (Fondazione Istituto Carlo Vismara - Giovanni De Petri Onlus, San Bassano/CR), **Michela Tovo** (Ospedale San Bassiano, Bassano del Grappa/VI), **Chiara Trattenero** (Centro Servizi Sociali Villa Serena - Residenza Fontanelle di Trissino/VI, Residenza Giardino di Recoaro Terme/VI, Villa Serena di Valdagno/VI), **Alessia Travanut** (Ospedale Santa Maria della Misericordia, Udine), **Donatella Tripodi** (RSA Alchemica, Torino), **Michela Uberti** (APSP Giudicarie Esteriori, Bleggio Superiore/TN), **Angelo Vacca** (Ospedale Giovanni XXIII - Policlinico di Bari), **Matteo Vacca** (Ospedale Sirai, Carbonia/SU), **Filippo Valbusa** (IRCCS Ospedale Sacro Cuore Don Calabria, Negrar/VR), **Fabrizio Vallelonga** (IRCCS Istituto Oncologico di Candiolo/TO), **Carla Vincenza Vallone** (Ospedale Umberto I, Nocera Inferiore/SA), **Giulia Vannini** (Ospedale San Giuseppe, Empoli/FI), **Edoardo Varratta** (Fondazione Policlinico Universitario Agostino Gemelli IRCCS, Roma), **Federica Vecchi** (Ospedale di Sassuolo/MO), **Carmen Vedele** (Policlinico di Sant'Orsola - IRCCS Azienda Ospedaliero-Universitaria di Bologna), **Grazia Ventisette** (RSA Pietro Beretta, Gardone Val Trompia/BS), **Francesca Venturelli** (Ospedale Villa Igea, Modena), **Lara Verdi** (Ospedale San Leopoldo Mandic, Merate/LC), **Ilaria Vergine** (IRCCS Ospedale San Raffaele, Milano), **Giovanna Vesnaver** (Ospedale Michele e Pietro Ferrero, Verduno/CN), **Patrizia Vidotto** (Centro Servizi per Anziani Nazaret, Zelarino/VE), **Valeria Villa** (Ospedale San Rocco, Sessa Aurunca/CE), **Fabio Villeggia** (Azienda Ospedaliera San Giovanni Addolorata, Roma), **Agostino Virdis** (Azienda Ospedaliero Universitaria Pisana, Pisa), **Antonella Visconti** (Ospedale San Paolo, Savona), **Sonia Visonà** (Centro Servizi Sociali Villa Serena - Residenza Fontanelle di Trissino/VI, Residenza Giardino di Recoaro Terme/VI, Villa Serena di Valdagno/VI), **Cinzia Vivarelli** (Ospedale Santa Maria della Misericordia, Udine), **Ana Vochescu** (Ospedale San Carlo Borromeo, Milano), **Stefano Volpato** (Azienda Ospedaliero-Universitaria di Ferrara), **Alfredo Vozza** (Ospedale Centrale Santissima Annunziata, Taranto), **Inis Vrusho** (Centro Servizi Contarini, Mestre/VE), **Giusy Vurchio** (Ospedale Centrale Santissima Annunziata, Taranto), **Alberto Zaccardelli** (Ospedale Santa Scolastica, Cassino/FR), **Giacomo Zaccherini** (Policlinico di Sant'Orsola - IRCCS Azienda Ospedaliero-Universitaria di Bologna), **Nicoletta Zaffarana** (Ospedale San Giovanni di Dio, Firenze), **Gloria Zagallo** (Centro Servizi per Anziani Nazaret, Zelarino/VE), **Giulia Zanella** (Centro Servizi Sociali Villa Serena - Residenza Fontanelle di Trissino/VI, Residenza Giardino di Recoaro Terme/VI, Villa Serena di Valdagno/VI), **Elisa Zanello** (Presidio Ospedaliero di Palmanova/UD), **Michela Zanetti** (Ospedale Maggiore, Trieste), **Samuel Zanetti** (CSA Villa Althea, Spinea/VE - CSA Villa Fiorita, Spinea/VE), **Luca Zanlari** (Ospedale della Val d'Arda, Fiorenzuola d’Arda/PC), **Elena Zanlorenzi** (Ospedale San Valentino, Montebelluna/TV), **Marco Zaramella** (Presidio Ospedaliero di Latisana/UD), **Chiara Zarivi** (Ospedale San Salvatore, L'Aquila), **Maria Beatrice Zazzara** (Fondazione Policlinico Universitario Agostino Gemelli IRCCS, Roma), **Raffaele Zoratti** (Presidio Ospedaliero di Palmanova/UD), **Benedetta Zuaboni** (Fondazione Angelo Passerini Onlus, Nozza di Vestone/BS), **Massimo Zuin** (Azienda Ulss 3, Venezia).

**Table 1a. Characteristics of internal medicine and geriatric wards and patients**

|  | Hospital | |
| --- | --- | --- |
|  | Internal Medicine | Geriatrics |
| **Ward/facility characteristics** |  |  |
| N of participating wards | 100 | 56 |
| N of beds, Median (IQR) | 33 (26–45) | 25 (20 − 39) |
| Availability of Palliative Care service/consultation (%) | 62 (62.0%) | 36 (64.3%) |
|  | | |
| **Patients/residents characteristics** |  |  |
| N of participating patients | 2,158 | 1,145 |
| Age, median (IQR) | 79 (69–86) | 85 (79–89) |
| Female gender (%) | 1,012 (46.9%) | 601 (52.5%) |
| Heart disease (%) | 853 (39.5%) | 504 (44.0%) |
| Cancer (%) | 667 (30.9%) | 264 (23.1%) |
| Cerebrovascular disease (%) | 322 (14.9%) | 252 (22.0%) |
| Dementia (%) | 512 (23.7%) | 433 (37.8%) |
| Other neurodegenerative disease (%) | 109 (5.1%) | 69 (6.0%) |
| Diabetes (%) | 581 (26.9%) | 297 (25.9%) |
| Pulmonary disease (%) | 535 (24.8%) | 279 (24.4%) |
| Liver disease (%) | 205 (9.5%) | 50 (4.4%) |
| Peripheral vascular disease (%) | 275 (12.7%) | 150 (13.1%) |

IQR=InterQuartile Range

**Table 2a. Palliative care needs assessed by NECPAL tool**

|  | Hospital | | Hospital  (n = 3,303) | Long term care facility  (n = 2,086) |
| --- | --- | --- | --- | --- |
|  | Internal Medicine  (n = 2,158) | Geriatrics  (n = 1,145) |  |  |
| **Whole sample** |  |  |  |  |
| Positive NECPAL | 1,243 (57.6%) | 681 (59.5%) | 1,924 (58.3%) | 1,003 (48.1%) |
| Positive SQ + ≥ 2 indicator | 1,176 (54.5%) | 659 (57.6%) | 1,835 (55.6%) | 936 (44.9%) |
| Positive SQ + ≥ 3 indicators | 1,044 (48.4%) | 592 (51.7%) | 1,636 (49.5%) | 697 (33.4%) |
| Positive SQ + ≥ 4 indicators | 811 (37.6%) | 478 (41.7%) | 1,289 (39.0%) | 460 (22.1%) |
| **Patients/residents with cancer** |  |  |  |  |
| Positive NECPAL | 513 (76.9%) | 206 (78.0%) | 719 (77.2%) | 112 (61.5%) |
| **Patients/residents without cancer** |  |  |  |  |
| Positive NECPAL | 730 (49.0%) | 475 (53.9%) | 1,205 (50.8%) | 891 (46.8%) |
| **Patients/residents aged < 80 y** |  |  |  |  |
| Positive NECPAL | 510 (45.3%) | 134 (45.0%) | 644 (45.3%) | 162 (32.3%) |
| **Patients/residents aged ≥ 80 y** |  |  |  |  |
| Positive NECPAL | 733 (71.0%) | 547 (64.6%) | 1,280 (68.1%) | 841 (53.1%) |
| **Men** |  |  |  |  |
| Positive NECPAL | 657 (57.3%) | 320 (58.8%) | 977 (57.8%) | 274 (45.1%) |
| **Women** |  |  |  |  |
| Positive NECPAL | 586 (57.9%) | 361 (60.1%) | 947 (58.7%) | 729 (49.3%) |

SQ=Surprise question

**Table 3a. Palliative care needs in patients admitted to internal medicine and geriatric wards**

|  | Hospital | |
| --- | --- | --- |
|  | Internal Medicine  n = 2,158 (%) | Geriatrics  n = 1,145 (%) |
| Dysphagia | 314 (14.6%) | 265 (23.2%) |
| Any pressure ulcer | 273 (12.8%) | 211 (18.6%) |
| Pressure ulcer (stage III-IV) | 88 (4.1%) | 61 (5.3%) |
| Functional decline* | 1,152 (53.4%) | 703 (61.4%) |
| Nutritional decline¶ | 764 (35.4%) | 445 (38.9%) |
| Repeated hospitalizations§ | 1,013 (46.9%) | 484 (42.3%) |
| Delirium # | 341 (16.7%) | 254 (23.8%) |
| Any pain¥ | 1,025 (47.5%) | 565 (49.3%) |
| Moderate to severe pain¥ | 587 (27.2%) | 269 (23.5%) |

* loss of > 30% in Barthel Index score over the previous 6 months

¶ unintentional loss of > 10% body weight over the previous 6 months

§ ≥2 hospital admissions within the last 12 months

\# 4AT score ≥ 4. Data on delirium were available for 2,037 patients in internal medicine wards (94.4% of study sample) and 1,068 patients in geriatric wards (93.3%)

¥ Any pain is defined as Numeric Pain Rating Scale ≥ 1 or Pain Assessment in Advanced Dementia ≥ 1; moderate to severe pain is defined as Numeric Pain Rating Scale ≥ 4 or Pain Assessment in Advanced Dementia ≥ 4
